# Decline in Receipt of Vaccines by Medicare Beneficiaries During the COVID-19 Pandemic — United States, 2020

**DOI:** 10.15585/mmwr.mm7007a4

**Published:** 2021-02-19

**Authors:** Kai Hong, Fangjun Zhou, Yuping Tsai, Tara C. Jatlaoui, Anna M. Acosta, Kathleen L. Dooling, Miwako Kobayashi, Megan C. Lindley

**Affiliations:** ^1^Immunization Services Division, National Center for Immunization and Respiratory Diseases, CDC; ^2^Division of Bacterial Diseases, National Center for Immunization and Respiratory Diseases, CDC; ^3^Division of Viral Diseases, National Center for Immunization and Respiratory Diseases, CDC.

On March 13, 2020, the United States declared a national emergency concerning the novel coronavirus disease 2019 (COVID-19) outbreak (*1*). In response, many state and local governments issued shelter-in-place or stay-at-home orders, restricting nonessential activities outside residents’ homes (*2*). CDC initially issued guidance recommending postponing routine adult vaccinations, which was later revised to recommend continuing to administer routine adult vaccines (*3*). In addition, factors such as disrupted operations of health care facilities and safety concerns regarding exposure to SARS-CoV-2, the virus that causes COVID-19, resulted in delay or avoidance of routine medical care (*4*), likely further affecting delivery of routine adult vaccinations. Medicare enrollment and claims data of Parts A (hospital insurance), B (medical insurance), and D (prescription drug insurance) were examined to assess the change in receipt of routine adult vaccines during the pandemic. Weekly receipt of four vaccines (13-valent pneumococcal conjugate vaccine [PCV13], 23-valent pneumococcal polysaccharide vaccine [PPSV23], tetanus-diphtheria or tetanus-diphtheria-acellular pertussis vaccine [Td/Tdap], and recombinant zoster vaccine [RZV]) by Medicare beneficiaries aged ≥65 years during January 5–July 18, 2020, was compared with that during January 6–July 20, 2019, for the total study sample and by race and ethnicity. Overall, weekly administration rates of the four examined vaccines declined by up to 89% after the national emergency declaration in mid-March (*1*) compared with those during the corresponding period in 2019. During the first week following the national emergency declaration, the weekly vaccination rates were 25%–62% lower than those during the corresponding week in 2019. After reaching their nadirs of 70%–89% below 2019 rates in the second to third week of April 2020, weekly vaccination rates gradually began to recover through mid-July, but by the last study week were still lower than were those during the corresponding period in 2019, with the exception of PPSV23. Vaccination declined sharply for all vaccines studied, overall and across all racial and ethnic groups. While the pandemic continues, vaccination providers should emphasize to patients the importance of continuing to receive routine vaccinations and provide reassurance by explaining the procedures in place to ensure patient safety (*3*).

Medicare enrollment and insurance claims data for beneficiaries enrolled in a fee-for-service plan during weeks 2–29 of 2019 (January 6–July 20) and 2020 (January 5–July 18) were obtained from the Centers for Medicare & Medicaid Services Chronic Conditions Warehouse (*5*). PCV13 and PPSV23 were covered by Part B, which pays for services from health care providers, outpatient care, and some preventive services. Td/Tdap was covered by Part B if it was administered as part of medically necessary service because of an injury and was covered by Part D, which covers a range of prescription drugs, if it was administered as part of preventive care. RZV was covered by Part D. Weekly claims for vaccination were identified by procedure code or national drug code on claims with a service date within the week examined (measured Sunday through Saturday).* Because some of the weeks spanned 2 different months, the denominator populations for PCV13 and PPSV23 vaccination in each week were defined as Medicare beneficiaries who were continuously enrolled in Parts A and B in the claim month and the previous month, and were aged ≥65 years on the first day of that previous month. The denominator population for Td/Tdap and RZV were defined similarly, except that beneficiaries were continuously enrolled in Parts A, B, and D. Weekly rates of receipt of the examined vaccines were calculated as the percentage of the denominator population that received ≥1 dose of the corresponding vaccine during that week.^†^ The percentage change in vaccination rate in a week was calculated as the ratio of the rate in that week in 2020 to the rate in the corresponding week in 2019, minus 1. Descriptive statistical analyses were conducted for the total study sample and stratified by race and ethnicity (non-Hispanic White [White], non-Hispanic Black [Black], Hispanic or Latino (Hispanic), non-Hispanic Asian/Asian American/Pacific Islander [Asian], non-Hispanic other [Other]).^§^ All statistical analyses were conducted during September 12–15, 2020, using SAS (version 9.4; SAS Institute). This activity was reviewed by CDC and was conducted consistent with applicable federal law and CDC policy.^¶^

During the study period, the average denominator populations were 27,194,802 in 2019 and 26,916,993 in 2020 for PCV13 and PPSV23, and 18,752,789 in 2019 and 18,701,076 in 2020 for Td/Tdap and RZV. Among Medicare beneficiaries, weekly rates of vaccination for each of the four vaccines declined precipitously after the national emergency declaration, compared with the corresponding weeks in 2019 (Figure). During January 5–March 14, 2020, weekly percentages of Medicare beneficiaries vaccinated with PPSV23, Td/Tdap, and RZV were consistently higher than were those during the corresponding week in 2019. Weekly vaccination rates dropped sharply during the first week after the national emergency declaration on March 13, with declines ranging from 25% for PPSV23 to 62% for RZV. The largest declines in weekly vaccination rates occurred during April 5–11, 2020, for PCV13, PPSV23, and Td/Tdap and during April 12–18, 2020, for RZV, when weekly vaccination rates dropped by 70% for Td/Tdap to 89% for RZV. After reaching this nadir, vaccination rates began to recover gradually. At the end of the study period (week commencing July 12), the weekly vaccination rate for PPSV23 was 8% higher than that during the corresponding week in 2019, but weekly vaccination rates for other examined vaccines remained 24% (Td/Tdap) to 43% (RZV) lower.

**FIGURE:**
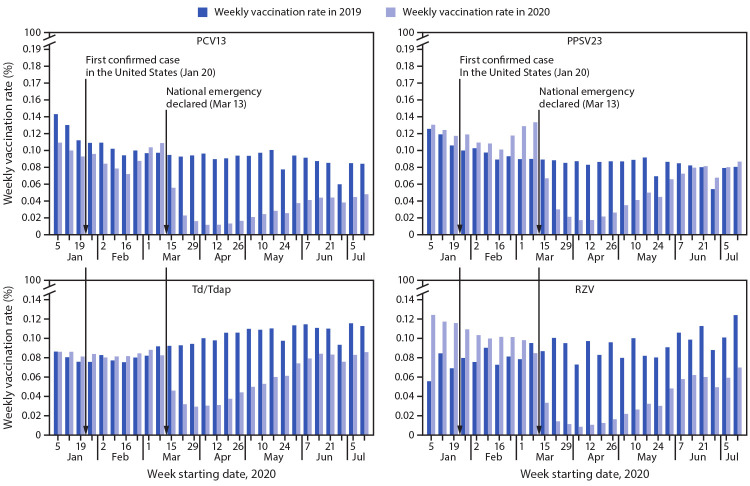
Percentage of Medicare beneficiaries aged ≥65 years who received PCV13,* PPSV23, Td/Tdap, and RZV^†^ vaccines, by week^§^ — United States, January 6–July 20, 2019^¶^ and January 5–July 18, 2020 **Source:** Medicare enrollment and claims data from the Centers for Medicare & Medicaid Services Chronic Conditions Warehouse. https://www2.ccwdata.org/web/guest/home **Abbreviations:** ACIP = Advisory Committee on Immunization Practices; PCV13 = 13-valent pneumococcal conjugate vaccine; PPSV23 = 23-valent pneumococcal polysaccharide vaccine; RZV = recombinant zoster vaccine; Td/Tdap = tetanus-diphtheria or tetanus-diphtheria-acellular pertussis vaccine. * ACIP voted to stop recommending routine PCV13 use among adults aged ≥65 years in June 2019. ^†^ In October 2017, RZV was approved by the Food and Drug Administration and recommended by ACIP preferentially over Zoster Vaccine Live for use in immunocompetent adults aged ≥50 years. ^§^ Calculated as the percentage of the study sample of Medicare enrollees who received ≥1 dose of the corresponding vaccine during that week. ^¶^ The starting date of the first examined week in 2019 is January 6.

Among Medicare enrollees in the study sample, an average (across all vaccines studied) of 85% were White, 7% were Black, 4% were Other racial and ethnic groups, and 2% each were Asian or Hispanic. Patterns of decline and recovery in vaccination among each racial/ethnic group were similar to the overall findings, but certain disparities in magnitude were observed (Table). By the most recently assessed week, the rate of PPSV23 receipt among White adults was 10% higher than that during the corresponding week in 2019, and the declines in PCV 13 and Td/Tdap vaccination (42% and 23%, respectively) were smallest among White adults. By comparison, the change in vaccination rates in the most recent week of the study period compared with the corresponding week in 2019 ranged from –57% (PCV13) to –9% (PPSV23) for Asian adults, –44% (PCV13) to –2% (PPSV23) for Black adults, –62% (PCV13) to –22% (PPSV23) for Hispanic adults, and –44% (RZV) to 3% (PPSV23) for Other race adults. The smallest decline in RZV vaccination rate (11%) during the most recent week was among Black adults.

**TABLE:** Change in weekly percentage* of Medicare beneficiaries aged ≥65 years who received routinely recommended adult vaccines, overall and by race/ethnicity^†^ — United States, January–July 2020

Vaccine^§^ and race/ethnicity	% Change in weekly vaccination rate^¶^		
	Prepandemic average Jan 5–Feb 29	Pandemic nadir**	Most recently assessed pandemic week July 12–18
**PCV13**			
**Total**	**−20**	**−88**	**−43**
White	−20	−88	−42
Black	−20	−87	−44
Hispanic/Latino	−20	−89	−62
Asian	−10	−92	−57
Other	−17	−89	−42
**PPSV23**			
**Total**	**12**	**−80**	**8**
White	12	−81	10
Black	9	−70	−2
Hispanic/Latino	10	−78	−22
Asian	22	−85	−9
Other	13	−83	3
**Td/Tdap**			
**Total**	**5**	**−70**	**−24**
White	5	−68	−23
Black	1	−76	−30
Hispanic/Latino	−3	−80	−44
Asian	11	−86	−38
Other	10	−76	−34
**RZV**			
**Total**	**47**	**−89**	**−44**
White	46	−89	−44
Black	57	−86	−11
Hispanic/Latino	65	−88	−40
Asian	58	−91	−53
Other	56	−89	−44

**Source:** Medicare enrollment and claims data from the Centers for Medicare & Medicaid Services Chronic Conditions Warehouse. https://www2.ccwdata.org/web/guest/home

**Abbreviations:** ACIP = Advisory Committee on Immunization Practices; PCV13 = 13-valent pneumococcal conjugate vaccine; PPSV23 = 23-valent pneumococcal polysaccharide vaccine; RZV = recombinant zoster vaccine; Td/Tdap = tetanus-diphtheria or tetanus-diphtheria-acellular pertussis vaccine.

* Calculated as the percentage of the study sample of Medicare enrollees who received ≥1 dose of the vaccine during that week. Percentage change in vaccination rate in a week was calculated as the ratio of the vaccination rate in that week in 2020 to the rate in the corresponding week in 2019, minus 1.

^†^ Black, White, Asian, and Other racial/ethnic groups were non-Hispanic; Hispanics could be of any race.

^§^ Weeks in 2020 were compared with the equivalent weeks in 2019: January 5–February 29, 2020 versus January 6–March 2, 2019 for prepandemic average; April 5–11 or April 12–18, 2020 versus April 7–13 or April 14–20, 2019 for nadir; July 12–18, 2020 versus July 14–20, 2019 for the last examined week.

^¶^ ACIP voted to stop recommending routine PCV13 use among adults aged ≥65 years in June 2019. In October 2017, RZV was approved by the Food and Drug Administration and recommended preferentially over Zoster Vaccine Live by ACIP for use in immunocompetent adults aged ≥50 years.

** Nadirs were observed during April 5–11 and April 12–18, 2020, depending on vaccine and race/ethnicity group.

## Discussion

Before the March 13, 2020, COVID-19 national emergency declaration, the weekly rate of receipt of PPSV23, Td/Tdap, and RZV** among Medicare beneficiaries aged ≥65 years in 2020 was consistently higher than that in the corresponding 2019 week. Because the Advisory Committee on Immunization Practices voted in June 2019 to stop recommending PCV13 for adults aged ≥65 years,^††^ vaccination with PCV13 among this population declined in 2020 compared with that in 2019. Since the declaration, rates of adult vaccination with these three vaccines and PCV13 were substantially lower than were those during the corresponding period in 2019, with steady recovery after mid-April 2020. These findings are consistent with previous reports of declines in routine pediatric vaccine ordering and vaccine administration (*6*) and childhood vaccination coverage (*7*) during the pandemic. Declines were similar across all racial/ethnic groups; however, the magnitude of recovery varied by race and ethnicity and vaccine. Vaccination rates among racial and ethnic minority adults were lower than were those among White adults.^§§^ The COVID-19 pandemic has disproportionally affected certain racial and ethnic minority groups directly (*8*); therefore, monitoring and early intervention to mitigate similar disparities in indirect effects of the pandemic, such as use of other preventive services, might be needed to avoid compounding this disparity.

The findings in this report are subject to at least three limitations. First, the analysis included only Medicare beneficiaries in a fee-for-service plan, which represents approximately 66% of total Medicare beneficiaries; therefore, the findings might not be applicable to all older U.S. adults, who might also have a different racial or ethnic distribution. Second, vaccination was identified on claims data submitted for reimbursement. Vaccination claims not accounted for were those that were not submitted yet or were not billed to Medicare. Finally, race/ethnicity groups other than White and Black could be potentially misidentified in the Medicare administrative enrollment records (*9*); therefore, actual declines in vaccination among those groups might be different from those reported.

Because all 50 states had begun lifting business restrictions or stay-at-home orders in some way by August 2020 (*10*), the likelihood of exposure to infectious diseases, including vaccine-preventable diseases, is increasing.^¶¶^ Levels of SARS-CoV-2 virus circulation and associated illnesses increased during September 2020–January 2021.*** In response, some jurisdictions reissued lockdown policies,^†††^ which might have affected observed recovery in vaccination rates. As the pandemic continues, vaccination providers should continue efforts to resolve disruptions in routine adult vaccination (*3*). When resuming in-person visits, vaccination providers should take actions to prevent the spread of SARS-CoV-2 and address patient concerns about exposure to SARS-CoV-2 during visits. Vaccination providers should also provide reassurance that vaccination services (including influenza vaccination to mitigate non-COVID respiratory illness and preserve health care capacity to treat COVID-19 during the influenza season) can be delivered safely and emphasize the importance of routine vaccination to protect patient health. It is important that vaccination providers counsel patients about expected reactogenicity of some vaccines, such as RZV, to help them understand the potential overlap between vaccination reactions and symptoms of COVID-19. Ultimately, continued efforts by vaccination providers and public health officials at all levels, including specific vaccination guidance for providers by state health departments,^§§§^ will be needed to ensure that routine adult vaccination returns to prepandemic levels to optimize protection of all older persons against vaccine-preventable diseases. Now that safe and effective COVID-19 vaccines are available, those efforts could also help older U.S adults obtain COVID-19 vaccination.

**Summary**

**What is already known about this topic?**

Routine health care services have been disrupted during the COVID-19 pandemic.

**What is added by this report?**

During the first week after the national COVID-19 emergency declaration in March 2020, weekly vaccination rates among Medicare beneficiaries aged ≥65 years declined by 25%–62%, compared with the corresponding period in 2019. By mid-April, vaccination rates in this group reached nadirs of 70%–89% below 2019 rates. Rates partially recovered gradually during May–July 2020.

**What are the implications for public health practice?**

Vaccination providers should emphasize the importance of routine adult vaccination to their patients and ensure the safe provision of vaccines to protect older adults from vaccine-preventable diseases during the ongoing COVID-19 pandemic.

## Footnotes

* Procedure codes alone for PCV13 and PPSV23 and procedure codes plus national drug codes for Td/Tdap and RZV.

† RZV was recommended by the Advisory Committee on Immunization Practices (ACIP) to be administered as a two-dose series. In the analysis a Medicare beneficiary was counted as receiving RZV if either the first or the second dose was administered and was counted twice if both doses were administered (in different weeks) during the study period. https://www.cdc.gov/mmwr/volumes/67/wr/mm6703a5.htm?s_cid=mm6703a5_w

§ Race/ethnicity of beneficiary was reported in the Medicare enrollment database, which was obtained from the Social Security Administration’s beneficiary record. American Indian or Alaska Native persons and persons whose race/ethnicity was unknown were grouped into Other. https://www2.ccwdata.org/documents/10280/19022436/codebook-mbsf-abcd.pdf

¶ 45 C.F.R. part 46, 21 C.F.R. part 56; 42 U.S.C. Sect. 241(d); 5 U.S.C. Sect. 552a; 44 U.S.C. Sect. 3501 et seq.

** The prepandemic weekly RZV vaccination rates in 2020 were notably higher than those in the equivalent week of 2019, likely because RZV was recently approved and recommended: In October 2017, RZV was approved by the Food and Drug Administration and recommended preferentially over Zoster Vaccine Live by ACIP for use in immunocompetent adults aged ≥50 years. https://www.cdc.gov/mmwr/volumes/67/wr/mm6703a5.htm?s_cid=mm6703a5_w

†† On June 26, 2019, ACIP voted to stop recommending routine PCV13 use among adults aged ≥65 years. Instead, ACIP recommended administration of PCV13 based on shared clinical decision-making for adults aged ≥65 years who do not have an immunocompromising condition, cerebrospinal fluid leak, or cochlear implant, and who have not previously received PCV13. https://www.cdc.gov/mmwr/volumes/68/wr/mm6846a5.htm?s_cid=mm6846a5_w

§§
https://www.cdc.gov/vaccines/imz-managers/coverage/adultvaxview/pubs-resources/NHIS-2017.html

¶¶ Persons can contract varicella zoster virus, the virus that causes zoster, from someone who has zoster. If the infected persons never had chickenpox or never received the chickenpox vaccine, they will develop chickenpox, and perhaps then develop zoster later in life. However, zoster cannot be contracted from someone who has zoster. https://www.cdc.gov/shingles/about/transmission.html

***
https://www.cdc.gov/coronavirus/2019-ncov/covid-data/pdf/covidview-02-05-2021.pdf

†††
https://www.chicago.gov/content/dam/city/sites/covid/health-orders/2021/2021.1.20-Stay-at-Home-Advisory.pdf

§§§ Washington: https://www.doh.wa.gov/Portals/1/Documents/1600/coronavirus/PleaseContinueVaccinatingPatients.pdf; Massachusetts: https://www.mass.gov/doc/encouraging-vaccination-during-the-covid-19-pandemic/download

## References

[1] Federal Emergency Management Agency. Bringing resources to state, local, tribal & territorial governments. Washington, DC: US Department of the Homeland Security, Federal Emergency Management Agency; 2020. https://www.fema.gov/disasters/coronavirus/governments

[2] Financial Industry Regulatory Authority. State “shelter-in-place” and “stay-at-home” orders. Washington, DC: Financial Industry Regulatory Authority; 2020. https://www.finra.org/rules-guidance/key-topics/covid-19/shelter-in-place

[3] CDC. Interim guidance for routine and influenza immunization services during the COVID-19 pandemic. Atlanta, GA: US Department of Health and Human Services, CDC; 2020. https://www.cdc.gov/vaccines/pandemic-guidance/index.html

[4] Czeisler MÉ, Marynak K, Clarke KEN, Delay or avoidance of medical care because of COVID-19–related concerns—United States, June 2020. MMWR Morb Mortal Wkly Rep 2020;69:1250–7. 10.15585/mmwr.mm6936a4 32915166 PMC7499838

[5] Centers for Medicare & Medicaid Services. CCW Medicare administrative data user guide, version 3.6. Baltimore, MD: US Department of Health and Human Services, Centers for Medicare & Medicaid Services; 2019. https://www2.ccwdata.org/documents/10280/19002246/ccw-medicare-data-user-guide.pdf

[6] Santoli JM, Lindley MC, DeSilva MB, Effects of the COVID-19 pandemic on routine pediatric vaccine ordering and administration—United States, 2020. MMWR Morb Mortal Wkly Rep 2020;69:591–3. 10.15585/mmwr.mm6919e2 32407298

[7] Bramer CA, Kimmins LM, Swanson R, Decline in child vaccination coverage during the COVID-19 pandemic—Michigan Care Improvement Registry, May 2016–May 2020. MMWR Morb Mortal Wkly Rep 2020;69:630–1. 10.15585/mmwr.mm6920e1 32437340

[8] Webb Hooper M, Nápoles AM, Pérez-Stable EJ. COVID-19 and racial/ethnic disparities. JAMA 2020;323:2466–7. 10.1001/jama.2020.8598 32391864 PMC9310097

[9] Zaslavsky AM, Ayanian JZ, Zaborski LB. The validity of race and ethnicity in enrollment data for Medicare beneficiaries. Health Serv Res 2012;47:1300–21. 10.1111/j.1475-6773.2012.01411.x 22515953 PMC3349013

[10] Lee JC, Mervosh S, Avila Y, See how all 50 states are reopening (and closing again). New York Times. August 10, 2020. https://www.cleansg.com/csg-blog-news/see-how-all-50-states-are-reopening-and-closing-again/

